# Ethnobotany of the Monpa ethnic group at Arunachal Pradesh, India

**DOI:** 10.1186/1746-4269-7-31

**Published:** 2011-10-14

**Authors:** Nima D Namsa, Manabendra Mandal, Sumpam Tangjang, Subhash C Mandal

**Affiliations:** 1 Department of Molecular Biology and Biotechnology, Tezpur University, Assam 784 028, India; 2 Department of Microbiology and Cell Biology, Indian Institute of Science, Bangalore 560 012, India; 3 Department of Botany, Rajiv Gandhi University, Rono Hills, Doimukh, Arunachal Pradesh 791112, India; 4 Division of Pharmacognosy, Department of Pharmaceutical Technology, Jadavpur University Kolkata 700 032, India

**Keywords:** Kalaktang Monpa, Ethnobotany, Medicinal plants, Arunachal Pradesh

## Abstract

**Background:**

The present paper documents the uses of plants in traditional herbal medicine for human and veterinary ailments, and those used for dietary supplements, religious purpose, local beverage, and plants used to poison fish and wild animals. Traditional botanical medicine is the primary mode of healthcare for most of the rural population in Arunachal Pradesh.

**Materials and methods:**

Field research was conducted between April 2006 and March 2009 with randomly selected 124 key informants using semi-structured questionnaire. The data obtained was analyzed through informant consensus factor (F_IC_) to determine the homogeneity of informant's knowledge on medicinal plants.

**Results:**

We documented 50 plants species belonging to 29 families used for treating 22 human and 4 veterinary ailments. Of the medicinal plants reported, the most common growth form was herbs (40%) followed by shrubs, trees, and climbers. Leaves were most frequently used plant parts. The consensus analysis revealed that the dermatological ailments have the highest F_IC _(0.56) and the gastro-intestinal diseases have F_IC _(0.43). F_IC _values indicated that there was high agreement in the use of plants in dermatological and gastro-intestinal ailments category among the users. *Gymnocladus assamicus *is a critically rare and endangered species used as disinfectant for cleaning wounds and parasites like leeches and lice on livestocks. Two plant species (*Illicium griffithii *and *Rubia cordifolia*) are commonly used for traditional dyeing of clothes and food items. Some of the edible plants recorded in this study were known for their treatment against high blood pressure (*Clerodendron colebrookianum*), diabetes mellitus (*Momordica charantia*), and intestinal parasitic worms like round and tape worms (*Lindera neesiana*, *Solanum etiopicum*, and *Solanum indicum*). The Monpas of Arunachal Pradesh have traditionally been using *Daphne papyracea *for preparing hand-made paper for painting and writing religious scripts in Buddhist monasteries. Three plant species (*Derris scandens*, *Aesculus assamica*, and *Polygonum hydropiper*) were frequently used to poison fish during the month of June-July every year and the underground tuber of *Aconitum ferrox *is widely used in arrow poisoning to kill ferocious animals like bear, wild pigs, gaur and deer. The most frequently cited plant species; *Buddleja asiatica *and *Hedyotis scandens *were used as common growth supplements during the preparation of fermentation starter cultures.

**Conclusion:**

The traditional pharmacopoeia of the Monpa ethnic group incorporates a myriad of diverse botanical flora. Traditional knowledge of the remedies is passed down through oral traditions without any written document. This traditional knowledge is however, currently threatened mainly due to acculturation and deforestation due to continuing traditional shifting cultivation. This study reveals that the rural populations in Arunachal Pradesh have a rich knowledge of forest-based natural resources and consumption of wild edible plants is still an integral part of their socio-cultural life. Findings of this documentation study can be used as an ethnopharmacological basis for selecting plants for future phytochemical and pharmaceutical studies.

## Background

Medicinal plants have been used as sources of medicine in many indigenous communities throughout the world. According to WHO, herbal medicines serve the health needs of about 80% of the world's population, especially for millions of people in the rural areas of developing countries [1]. India has a rich source of medicinal plants distributed in different geographical conditions and the large sections of Indian population still rely on traditional plant medicines as they are abundantly available, economical, and have little or no side-effects in addition to their cultural acceptability [2-4]. The plant-based knowledge, largely oral, has been transferred from one generation to the next through traditional healers, knowledgeable elders or ordinary people without any written documents. We found that the indigenous knowledge on plant resources was confined to elder members of the study area and the younger's have little or no contribution in this aspect. The study of ethno-botanical plants provides an opportunity for multi-disciplinary and interdisciplinary research work between botany, pharmacology and toxicology, chemistry, anthropology and sociology. The total population of the Arunachal spreading over 16 districts is about 1,019,177 (Population census, 2001), is home to about 28 major tribes and 110 sub-tribes [5]. Each district has its own composition of tribes with distinctive dialects, custom, traditional beliefs and cultural diversity. Medicinal plants have been used as sources of traditional medicine in virtually all tribal cultures and today, according to World Health Organization as many as 80% of the world's population depend on traditional medicine for their primary healthcare needs. In Arunachal, about 5000 species of angiosperms has been recorded and over 500 species of plants are used in the traditional healthcare system to treat various ailments [6]. Herbal plants use for the preparation of Ayurvedic, Unani, Sidha and homoeopathic medicines are available in different climatic zones of the state [7]. In addition to tribal medicines, plants and their parts are commonly used as food supplements, dying clothes, veterinary health care, handicrafts, rituals, local beverage (beer) production, seasonal fishing, and hunting [8-11]. The existence and dependency on a large number of traditional practices can be thought of as an alternative type of medicine, where the cost and side effects are negligible. Doley *et al *[12] reported a unique medicinal plant uses among the Nyishi community of Arunachal Pradesh. The consumption of wild edible plants are used as supplements to cultivated crops and as a survival strategy during food shortages that appears to have been intensified due to low development of agricultural production. Tag and Das [11] documented the ethnobotanical importance of 28 plants species, which are particularly used as food, medicine, in rituals and other ethnobotanical importance of the Hills Miri tribe of Arunachal Pradesh. Deb *et al *[7] while studying the Nyishi ethnic community of Arunachal Pradesh reported that a large number of traditional crops grown in agro-forestry are valuable for the farmers' everyday life, as they provide a greater diversity of food and also act as a good source of commercial outlets in addition to household consumption. They also reported the importance of plant species like bamboo, *Areca catechu *and *Livistonia jenkinsiana *that are useful for fencing, craft making, house construction and valued for traditional worship as they are associated with ancestral sacrifices. Goswami *et al *[13] reported a total of 10 medicinal plants used by the Tagin tribe of Arunachal Pradesh for the treatment of common illness as well as for ethno-veterinary use. Utilization of this traditional knowledge of medicinal plants is not only useful for conservation of cultural traditions and biodiversity but also for community healthcare and drug development. Srivastava *et al *[14] reported a total of 106 plants species used in food, medicine, hunting, cultural and handicrafts by the Apatani tribe. Kagyung *et al *[15] reported a total of 44 plant species used by Adi tribe of Arunachal Pradesh for the treatment of various gastro-intestinal diseases. Sen et *al *[16] documented the traditional herbal knowledge of Khampti tribe of Arunachal and found the highest number of species used for treatment of lung related diseases. Sarmah *et al *[17] reported a total of 63 medicinal plant species used by Chakma community of Arunachal Pradesh for the treatment of common diseases such as diarrhea, malaria, cough, dysentery, and gastro-intestinal disorders. Dutta and Bhattacharjya [18] have studied an indigenous community fishing practiced by the *Wancho *tribe of Tirap district, Arunachal Pradesh.

Although the rich indigenous knowledge on the medicinal use of plants has been relatively well documented in other ethnic groups of Arunachal Pradesh [12-21], studies on the knowledge of medicinal and wild edible plants of Kalaktang Monpa are limited. In previous study, we reported that the Lohit community of Arunachal Pradesh have a rich knowledge on herbal remedies for treating inflammation-related diseases [21] and different tribes inhabiting in the state has a rich reservoir of traditional knowledge on natural resources.. The most serious threat to the existing knowledge and practice on traditional medicinal plants included cultural change, particularly the influence of modernization, lack of written document, deforestation, environmental degradation, and lack of interests shown by the next younger generations were the main problems reported by the informants during the field survey. Urgent ethno-botanical studies and subsequent conservation measures are required to prevent the loss of valuable indigenous knowledge of medicinal plants of several indigenous communities in Arunachal Pradesh. In the absence of modern rural link road and the lack of infrastructure in sub-health center in each villages covered in this study, the tribal communities primarily rely on plant-based remedies to meet their basic healthcare needs. Therefore, the assessment and documentation of ancestral knowledge of indigenous people on traditional plant medicines would fill the gap associated knowledge between the elders and the younger generation on medicinal plants. The purpose of this ethno-botanical study was to present the results of ethno-botanical field survey conducted between April 2006 and March 2009, which was analyzed with two different quantitative ethno-botanical tools to select the important species used in traditional medicine and the homogeneity of indigenous knowledge amongst Monpa ethnic group of Kalaktang, Arunachal Pradesh, India.

## Materials and methods

### Study area: Kalaktang

**Figure 1:**
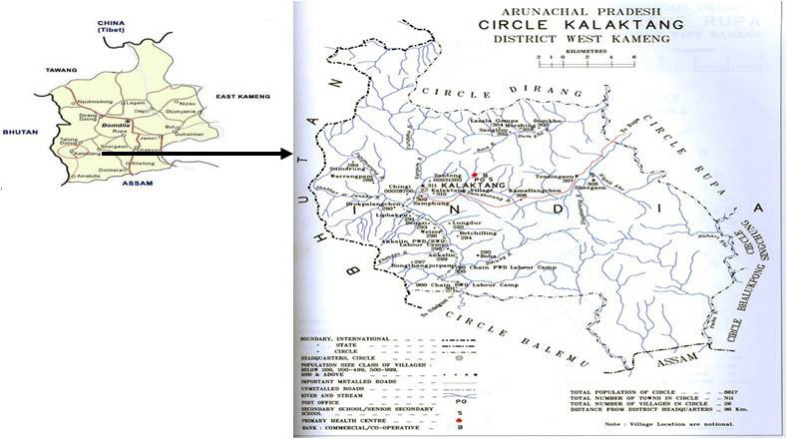
**A detailed study map of study area Kalaktang showing the geographical locations of villages covered during the field work**.

The Kalaktang region (417 sq. km.) is located approximately between 91° 30'-92° 40' East longitudes and 26° 54'-28° 01' North latitudes with an estimated total population of 6,391 (male 3,318, female 3,073, total literacy 60.37%, male 69.87% and female 48.12%, Population Census, 2001). It shares an international border with the Tibet region of China in the North, Bhutan in the West, Tawang district and East Kameng districts of Arunachal are in the northwest and northeast, respectively. The southern boundary adjoins the Sonitpur district of Assam (Figure 1). The general topography of the region falls within the higher mountainous zone, cluster of tangled peaks and valleys intercepted by two major riverbeds- Nargum and Domkhorong and its large number of tributaries. The altitude of Kalaktang region is at 1113 m above sea level, the minimum and maximum temperature recorded ranged from 17.0°C to 31.5°C, respectively. The average humidity is relatively high (75%-80%) during June-July months and it receives annual average rainfall of 674.50 mm. Black, red, sandy and clay type soil predominate the entire study area. The vegetation of the area comprises of semi-evergreen, evergreen, deciduous, moist and temperate forests. The Kalaktang region comprises of 25 villages with only one community health center located at Kalaktang, a sub-center in each village covered in the present field work and a few villages are connected by a rural link road.

### Ethnology and cultural background: Monpa tribe

The west kameng district is inhabited by five different tribes such as the Akas, Khowas, Mijis, Sherdukpens, and Monpa. The entire population of the west kameng district can be divided into two cultural groups on the basis of their socio-religious affinities, of which the Monpas and Sherdukpens follow the lamaistic tradition of Mahayana Buddhism. The second groups of the people are Akas, Mijis, and Buguns, who worship the Sun and the Moon as God, locally called as "Donyi-Polo" and "Abo-Tani", respectively. Due to slight variations in dialects, Monpa can be divided into six linguistic groups, namely Tawang Monpa, Dirang Monpa, Lish Monpa, Boot Monpa, Panchen Monpa, and Kalaktang Monpa. The Monpa have castes and clans with no social hierarchy. Monogamy (follow strictly endogamy) is a general rules though polygamy is also practiced in the present generation. The Monpa belongs to the Tibeto-Mongoloid racial stock and their houses are built of stones and timber decorated with a small altars and chapels with statues of Lord Buddha. Offering water in seven little cups and burning butter lamps and some leaves of herbal species (*Pinus wallichiana *A.B. Jackson, *Pinus longifolia *Roxb. and *Thuja occidentalis *L.) are daily rituals. They believe in transmigration of soul and reincarnation. The Monpas perform many pantomime dances of which "*Achilamu*", a group of five member dance is the most unique and popular form of dance perform throughout the day to complete the process in special occasions. Festival forms essential aspects of socio-religious life of the Monpas. Lossar and Choskar are the major religious festival of Monpa celebrated once in a year. Lossar, usually celebrated in the month of March before the start of agriculture is the local new year of the Monpa community. In Choskar festival (celebrated after sowing crops like maize, paddy, etc), the lamas or Monks read religious scriptures in the Gonpa (monastery) for a number of days (3-4 days). Thereafter, the villager's particularly female folk (both married and unmarried) carry the religious books on their back in the procession under the guidance of senior most Monk and the procession (1 day) covers throughout the cultivation fields. The significance of this performance is to ensure bumper harvest and crop/grains protection from insects and wild animals and for overall prosperity of the village people. The Monpas are agriculturist, practice both shifting and permanent types of cultivation. The commonly grown field crops include maize, paddy, beans, bajra, millets, barley, wheat, mustard, cabbage, potato, cauliflower, and pumpkin, etc. Livestock's like yaks, cows, pigs, sheep, seasonal fishing, and hunting of wild animals are the primary source of income. The Monpas are well known for wood curving, painting religious scrolls called Thankas, carpet and paper making, and weaving.

### Ethno-botanical survey and consensus analysis

A total of 27 field visits (8-10 days in each survey) were conducted amongst Monpa community during the study period from April 2006 and March 2009 to document an indigenous traditional knowledge on medicinal plants. Male and female respondents with age ranging from 20-60 years were included during interview. All collections were made by the first author (NDN) who grew up and belonged to Monpa community of Kalaktang and was familiar with the local language and some of the traditional plants used by the local people of the region. The ethno- botanical information was collected using semi-structured questionnaires [21,22] to address the following objectives:

1. Document the medicinal plants used in the traditional healthcare system of Kalaktang study area-parts used and method of preparation,

2. The informant consensus factor (F_IC_) was calculated in order to estimate use variability of medicinal plants,

3. Reliability of medicinal plant was assessed by comparing indigenous plant use with online literature reports on phytochemical and pharmacological properties,

4. How is the traditional knowledge of indigenous people preserved, utilized and transmitted to next generation?

Only the plants indicated by at least 20 independent informants were considered. The acquired data were confirmed by repeated queries and field surveys made among the general local people, experienced elderly people and 20 male respondents that constitute the traditional herbal practitioners. The taxonomic identification of the collected plant specimens was made with the help of herbarium materials, experts and taxonomic keys at Botanical Survey of India, Arunachal Pradesh. The botanical nomenclature followed that of the Flora of Arunachal Pradesh [23-25]. The voucher specimens were deposited in the Department of Molecular Biology and Biotechnology, Tezpur University for future reference. Calculation of a consensus factor (F_IC_) for testing homogeneity on the informant's knowledge was followed by the method provided by Trotter and Logan [26]. A consensus factor of F_IC _is given by:

$${\text{F}}_{\text{IC}}={\text{N}}_{\text{ur}}-{\text{N}}_{\text{t}}∕\left({\text{N}}_{\text{ur}}-\text{1}\right)$$

The factor provides a range of 0 to 1, where a high value acts as a good indicator for a high rate of informant consensus. N_ur _is the number of use-reports of informants for particular illness usage, where a use-report is a single record for use of a plant mentioned by an individual, and N_t _refers to the number of species used for a particular illness category for all informants. The use of "general categories" is adopted here as recommended by other ethnobotanical researchers [27,28]. These 22 illnesses were clustered into 4 usage (dermatological, gastro-intestinal, general health and miscellaneous disorders) categories (Table 1).

**Table 1:** Ethnobotanical consensus index for traditional medicinal plant use categories

Illness category (diseases and disorders)	**Number of Taxa (N**_**t**_**)**	**Number of use-reports (N**_**ur**_**)**	**Informants' consensus index factor (F**_**IC**_**)**^**a**^
Dermatological disorder (Scabies, skin diseases, pimples, eczema, inflammations, wound healing, cuts)	15	34	0.56
Gastro-intestinal disorder (Gastritis, diarrhea, dysentery, stomach ache, intestinal worms, and throat clearance)	21	36	0.43
General Health (Tooth ache, bone fracture, heart problem, cough, diabetes, high blood pressure, and jaundice)	9	11	0.20
Miscellaneous (Poison, veterinary diseases, beverages, rituals and religious, fodder, condiments, and soap)	24	29	0.17

^**a**^F_IC _= N_ur_-N_t_/(N_ur_-1), providing a value between 0 and 1, where high value indicates a high rate of informant consensus.

## Results and discussion

### Medicinal plants, growth forms and plant parts

This study identified fifty ethnobotanical species, 36 species (60%) were used as herbal medicines for treating 22 different human ailments. Some of the reported plants were used for other functions: rituals (14%) and religions, fish feeds and poisoning (10%), veterinary healthcare (7%) and local beverage or fermentation purpose (7%). The surveyed plants contained the following ethno-botanical elements: botanical name, voucher specimen number, local name, parts used, and method of preparations and ailments treated (Table 2). The families Asteraceae and Solanaceae had six and three species, respectively. Most of the ethnobotanical plants (50 species in this study) were herbs (40%), shrubs (28%), trees (26%), and climbers (6%). This study recorded that several parts of individual plant species were used as a medicine. The use of aerial plant parts (91%) was higher than the underground plant parts (9%). Leaves (53%) were predominantly used as a remedy followed by the fruit/seed/pod (26%), whole plant (9%), rhizome (6%), tuber (3%), and flower (3%). The preference for leaf has also been recorded amongst the Kani communities in India [29], the Malasars of Dravidian Tamils occupying the forests of the Western Ghats, South India [30] and the traditional Tibetan doctors (Amchi) of Mustang district of the north-central part of Nepal [31]. The common use of herbs as sources of medicine found in this study were also indicated by studies conducted elsewhere [29-32].

**Table 2:** Ethno-botanical uses of plants documented in the study area: Kalaktang, Arunachal Pradesh

Botanical name/Voucher number	Family name	Local name/Status of domestication	Habit	Parts used	Herbal formulation	Ailments treated
* Artemisia nilagirica *(Clarke) Pamp. (N/2005-20)	Asteraceae	Merangma, Wild	Sh	Leaves	Juice and paste (E)	Wounds, cuts, scabies, and inflammations
*Ageratum conyzoides *Linn. (N/2005-21)	Asteraceae	Ngonamshu, Wild	H	Leaves	Juice and paste (E)	Wound healer, Veterinary, fish poison
*Azadirachta indica *A. Juss (N/2006-2001)	Meliaceae	Neem, Wild	T	Leaves	Decoction (I)	Stomach disorder, diarrhoea
*Allium sativum *Linn. (N/2006-200)	Liliaceae	Chong, Cult	H	Leaves and rhizome	Paste and juice (E)	Bone fracture
*Allium hookeri *Linn. (N/2005-22)	Liliaceae	Lam, Cult	H	Leaves and rhizome	Paste and juice (E)	Skin diseases, Veterinary, bone fracture,
*Aesculus assamica *Griffith (N/2005-40)	Sapindaceae	Thretangshing, Wild	T	Stem bark	Fresh barks collected and pounded with wooden stick	Fish poison
*Aconitum ferox *Wall. (N/2005-46)	Ranunculaceae	Shaga-manshing, Wild	H	Tuber	Paste in arrow (made of iron) poisoning	Poison to kill rats and wild animals
*Bidens pilosa *Linn. (N/2006-222)	Asteraceae	Robashing, Wild	H	Leaves	Decoction and paste (E)	Wounds and skin inflammations
*Buddleja asiatica *Lour. (N/2006-223)	Scrophulariaceae	Phamshing, Wild	Sh	Leaves and young twigs	Juice and paste (E)	Diarrhoea, Beverages fermentation
*Cannabis sativa *Linn. (N/2005-19)	Urticaceae	Namku, Wild	Sh	Leaves and seeds	Mixed with maize flour	Veterinary
*Castanopsis indica *Roxb (N/2005-38)	Fagaceae	Kheshing, Wild	T	Leaves and stem bark	Whole plant extract is used to poison fish	Fish poison and raw seeds are eaten
*Curcuma caesia *Roxb. (N/2005-28)	Zingibaraceae	Yongka, Cult	H	Rhizome	Paste (E)	Rituals and pimples removal
*Centella asiatica *Linn. (N/2006-224)	Apiaceae	Manimuni, Cult	H	Whole plant	Decoction (I), vegetable	Stomach disorder, cuts, wounds, inflammations& common vegetable
*Clerodendrum colebrookianum *Walp. (N/2005-24)	Verbenaceae	Khangjela-shing, Wild	Sh	Leaves	Decoction with sugar (I), boiled vegetable	High blood pressure, stomach disorder, headache
*Citrus indica *Tanaka (N/2005-25)	Rutaceae	Tsalum, Cult	T	Fruit/seeds	Paste (E)	Face pimples removal
*Dioscorea alata *Linn. (N/2005-23)	Dioscoreaceae	Rangthangong, Cult	C	Tuber	Boiled vegetable	Gastritis
*Derris scandens *(Roxb.) Benth. (N/2005-32)	Leguminaceae	Sa-ngairushing, Wild	C	Roots	Roots are pounded with wooded stick and thrown into the river to poison fishes	Community fishing
*Ficus glomerata *Roxb. (N/2005-30)	Moraceae	Koknangshing, Wild	T	Fruits/seeds	Eaten raw	Diabetes and common fodder
*Gynura crepedioides *(BTH.) Moore (N/2006-225)	Asteraceae	Jakpangon, Wild	H	Leaves and young twigs	Boiled vegetable/raw	Vegetables and stomach disorder
*Gymnocladus assamicus *Kanjilal ex. P.C. Kanjilal (N/2005-17)	Fabaceae	Minangmashing, Wild	T	Mature pods	Bark	Detergent (soap), religious and veterinary
*Hedyotis scandens *Roxb. (N/2006-228)	Rubiaceae	Phamshing, Wild	Sh	Leaves and young twigs	Decoction with sugar (I)	Gastritis, Beverages fermentation
*Houttuynia cordata *Thunb. (N/2006-229)	Piperaceae	Momarengpa, Wild	H	Whole plant	Decoction (I)/boiled vegetable/raw	Stomachache and diarrhoea
						
*Ipomoea batatas *Linn. (Lam.) (N/2005-35)	Convolvulaceae	Yengjoktang, Cult	H	Leaves and tuber	Boiled vegetable	Rituals, tubers staple food and leaves as fish feeds
*Leucas aspera *Spreng. (N/2006-230)	Lamiaceae	Ngonshing, Wild	H	Leaves	Juice and paste (E)	Cuts and wounds, earache, inflammation
*Litsea cubeba *(Lour) Pers. (N/2005-38)	Lauraceae	Nengshing, Wild	T	Fruits	Paste (E), Raw/cooking	Condiments, eczema, heart disease and stomach disorder
*Lindera neesiana *(Wallich ex Nees) Kurz (N/2005-39)	Lauraceae	Lungkarmashing, Wild	T	Fruits/Seeds	Hot oils taken 2-3 spoonful (I)	Anthelmintic, diarrhoea, scabies, vegetable oils
*Momordica charantia *Linn. (N/2006-236)	Cucurbitacaea	Kairu, Cult	C	Fruit/Seeds	Cooking/raw	Anthelmintic, diabetes
*Mannihot esculenta *Crantz (N/2006-238)	Euphorbiaceae	Shingjoktang, Cult	Sh	Rhizome	Cooking	Rituals, vegetables
*Oroxylum indicum *Vent. (N/2006-240)	Bignoniaceae	Namkalingshing, Cult	T	Fruits/seeds	Seeds collected and dried	Rituals
*Ocimum sanctum *Linn. (N/2006-244)	Lamiaceae	Tilosi, Cult	H	Leaves	Paste (E), hot water decoction (I)	Stomach disorder, inflammations, wounds, cuts
*Pinus wallichiana *A.B. Jackson (N/2005-16)	Pinaceae	Chhu-gon-shing, Wild	T	Leaves and cones		Rituals and resins
*Pinus longifolia *Roxb (N/2005-15)	Pinaceae	Chhu-gon-shing, Wild	T	Leaves		Ritual
*Piper betle *Linn. (N/2005-14)	Piparaceae	Unknown, Cult	H	Leaves		Beverages fermentation
*Polygonum hydropiper *Linn. (N/2005-36)	Polygonaceae	Ngashing, Wild	H	Whole plant	Whole plant extract	Fish poison
*Psidium guajava *Linn. (N/2006-252)	Myrtaceae	Baghanse, Cult	T	Leaves	Raw/decoction with citrus fruit juice and salt (I)	Darrhoea, cough
*Punica granatum *Linn. (N/2005-37)	Punicaceae	Dalemshing, Cult	H	Leaves	Decoction	Stomach ache and diarrhoea
*Pouzolzia bennettiana *Wight (N/2005-52)	Urticaceae	Oyek, Wild	Sh	Leaves	Boiled vegetable	Stomach disorder
* Plantago major *Linn. (N/2005-42)	Plantaginaceae	Tsashing, Wild	H	Whole plant	Paste and juice (E)	Wounds, inflammations, Veterinary
*Rhododendron arboreum *Smith. Gurans (N/2005-49)	Ericaceae	Woodongmento, Wild	T	Flower	Decoction with sugar (I)	Dysentery, diarrhoea, throat clearance when fish bones get stuck in the gullet
*Solanum xanthocarpum *Burm. f. (N/2005-44)	Solanaceae	Zubalemin, Wild	H	Seeds	Paste (I)	Dental problem
*Solanum indicum *Linn. (N/2005-54)	Solanaceae	Kharangeh, Cult	Sh	Seeds	Boiled vegetable/raw	Anthelmintic, Beverages fermentation
*Solanum torvum *Sw. (N/2005-50)	Solanaceae	Borang Kharangjeh, Wild	Sh	Seeds	Boiled vegetable/raw	Anthelmintic
*Solanum sp*. (N/2005-13)	Solanaceae	Apataniseh, Wild	Sh	Seeds	Boiled vegetables/raw	Antihelminthic
*Saccharum officinarum *Linn. (N/2006-243)	Poaceae	Khumin, Cult	Sh	Stem	Juice (I)	Jaundice
*Spilanthes oleracea *Murr. (N/2006-246)	Asteraceae	Marshang, Wild	H	Leaves and young twigs	Paste (E)/boiled vegetable	Stop bleeding, skin infections and gastritis, fish poison
*Thysanolaena maxima *Kuntze (N/2006-250)	Poaceae	Tsakpushabashing, Wild	Sh	Whole plant	Whole plant collected and dried	Rituals
*Thuja occidentalis *Linn. (N/2005-12)	Cupressaceae	Pos-shing, Wild	Sh	Whole plant		Rituals
*Zingiber officinale *Rosc. (N/2005-48)	Zingiberaceae	Saagha,Cult	H	Rhizome	Raw/vegetable	Cough and Stomachache

Habit: T: tree; Sh: shrub; H: herb; C: climber; Cult: cultivated.

Mode of administration: (I) internal use; (E) External use.

### Consensus of traditional knowledge

This study indicates a high level of consensus within the Monpa ethnic community. In our study, the informant consensus of medicinal plant usage with the Monpa ethnic group resulted in F_IC _factors ranging from 0.17 to 0.56 per illness category (Table 1). The consensus analysis revealed that the category dermatological disorders have the highest F_IC _factor of 0.56 and the gastro-intestinal diseases have intermediate F_IC _(0.43), indicating greater homogeneity among informants. The highest F_IC _value for dermatological and gastro-intestinal diseases categories could be related to the high occurrence of skin-related and gastritis problems in the study area. The F_IC _of local knowledge for disease treatment depended on the availability of plant species and the occurrence of diseases in the study area. In the literature, high informant consensus (F_IC _0.875) was also recorded among the snakebite healers of Kamba in Africa [33] and treating 'mich' or febrile diseases (F_IC _0.80) among Northwestern Ethiopia [34]. The fidelity value (FL) of a plant species for a specific disease in the study area varied between 30 and 100%. The maximum FL of 100% expressed by *Artemisia nilagirica*, *Azadirachta indica*, *Allium sativum*, *Cannabis sativa*, *Clerodendrum colebrookianum*, *Gymnocladus assamicus*, *Lindera neesiana*, *Ocimum sanctum*, *Psidium guajava*, and *Saccharum officinarum*, for wound healing and scabies, stomach disorder and diarrhea, bone fracture, diarrhea in cattle, high blood pressure, soap and ethno-veterinary, intestinal worms, wounds, diarrhea, and jaundice, respectively, indicated the 100% choice of most healers or plant practitioners for treating such diseases. The literature search on ethnopharmacological use showed that many of the species of plants with 100% FL were used to treat ailments in other parts of the world (See Table 3). A specific example includes *Artemisia nilagirica *[35], *Azadirachta indica *[36], *Allium sativum *[37], *Clerodendrum colebrookianum *[38-40], *Gymnocladus assamicus *[41,42]*Lindera neesiana *[43], *Ocimum sanctum *[44], *Psidium guajava *[45-47], *Momordica charantia *[48,49], *and Rhododendron arboreum *[50]. However, the pharmacological properties of an individual plant can be significantly altered in the presence of other plant species in compound medicines. *Psidium guajava *is one of the most recorded plant species used to treat diarrhoea in developed countries [45]. On the other hand, the lowest FL of 30% indicated less preferred species by the traditional healers for treating specific ailment. For example, *Eupatorium adenophorum *was used for treating freshly cuts and wounds; *Houttuynia cordata *was used for treating stomach ache and diarrhea.

**Table 3:** Fidelity Level (FL) of interesting medicinal plants of the study area

Plants	Illness categories	Fidelity level (FL) (%)	Published related ethno-pharmacological references
*Artemisia nilagirica*	Wound healing, scabies	100	Antifungal activity [22]
*Azadirachta indica*	Stomach disorder, diarrhea	100	Antibacterial and antidiarrhoeal activity[23]
*Allium sativum*	Bone fracture	100	Anti-inflammatory activity [24]
*Cannabis sativa*	Diarrhea in cattle	100	
*Clerodendrum colebrookianum*	High blood pressure	100	Remedy for treatment of hypertension [25-27]
*Gymnocladus assamicus*	Soap, ethnoveterinary	100	Soap/detergent substitute [28,29]
*Lindera neesiana*	Intestinal worms	100	Essential oil [30]
*Ocimum sanctum*	Stomach disorder, wounds	100	Wound healing activity; Gastro-protective; Flavanoids [31]
*Psidium guajava*	Diarrhea	100	Antidiarrhoeal, Antibacterial activity [32-34]
*Saccharum officinarum*	Jaundice	100	
*Momordica charantia*	Intestinal worms, diabetes	80	Anti-diabetic activity, triterpenoids [35,36]
*Solanum xanthocarpum*	Dental problem	80	
*Rhododendron arboreum*	Diarrhea, throat clearance	78	Quercetin, rutin, coumaric acid [37]
*Plantago major*	Wounds, inflammations, ethnoveterinary	68.85	
*Zingiber officinale*	Cough and throat clearance	67	
*Ageratum conyzoides*	Wound healer	60	
*Solanum etiopicum*	Intestinal worms	56	
*Solanum indicum*	Intestinal worms	48	
*Eupatorium adenophorum*	Freshly cuts and wounds	30	
*Houttuynia cordata*	Stomach ache, diarrhea	30	

### Comparison of indigenous plant use with available pharmacological reports

An empirical observation on the use of medicinal plants by the Monpa people of Kalaktang study area requires cross-validation with published literatures on phytochemical and pharmacological properties of medicinal plants reported in this study to corroborate their bio-efficacy. Literature review for 27 medicinal plant species revealed that the reported local use was coherent with known pharmacological properties (See Table 4). Comparison of the information on traditional medicinal plant use of Monpa ethnic group with ethnobotanical studies conducted in other ethnic communities of Arunachal Pradesh [11-18] shows similar results for many species. This is of significance because identical plant use by several communities' from different areas may be a reliable indication of curative properties.

**Table 4:** Comparison of indigenous plant use and pharmacological properties of reported medicinal plants

Species name	Indigenous use	Reported phytochemical/pharmacological properties	Local use coherent with known phytochemical/pharmacological properties
*Artemisia nilagirica*	Wounds, scabies, inflammations	Anti-microbial, anti-fungal activity and polyphenolic compounds [64,65].	Yes
*Ageratum conyzoides*	Wound healer	Anti-inflammatory, analgesic, anti-pyretic, anti-microbial, and wound healing properties [66,67]. Tannins, saponins, coumarins, flavonoids, pyrrolizidine alkaloids [68].	Yes
*Azadirachta indica*	Stomachache, diarrhoea	Anti-inflammatory, anti-pyretic, analgesic, anti-ulcerogenic properties [69,70].	Yes
*Allium sativum*	Bone fracture	Anti-inflammatory in experimental rats [71].	Yes
*Bidens pilosa*	Wounds healer	Anti-inflammatory, anti-allergic activity (Horiuchi and Seyama, 2008; Inflammations, bacterial infections [72].	Yes
*Centella asiatica*	Stomach disorder, wounds	Anti-inflammatory activity, wound healing activity of asiaticoside [73]. Triterpenicconstituents asiaticoside, asiatic acid andmadecassic acid [74].	Yes
*Clerodendrum colebrookianum*	High blood pressure	Used for high blood pressure [75,76].	Yes
*Dioscorea alata*	Gastritis	No relevant report found	
*Ficus glomerata*	Diabetes	Hypoglycemic activity in alloxan-induced diabetic rats [77].	Yes
*Gynura crepedioides*	Stomach disorder	No relevant report found	
*Hedyotis scandens*	Gastritis	No relevant report found	
*Leucas aspera*	Wounds,earache, inflammation	Antimicrobial activity [78]. Leucasin and anti-oxidant activity [79].	Partial
*Litsea cubeba*	Eczema, stomach disorder	Fungicidal terpenoids and essential oil [80].	Partial
*Lindera neesiana*	Anthelmintic, diarrhoea	Essential oil and antimicrobial activity [81].	Partial
*Momordica charantia*	Anthelmintic, diabetes	Anti-diabetic activity [82].	Yes
*Ocimum sanctum*	Stomachache, inflammations, wounds	Anti-oxidant and wound healing activity [83]. Leaf paste applied on infected skin [84].	Yes
*Psidium guajava*	Diarrhoea, cough	Anti-diarrhoea activity [85].	Yes
*Punica granatum*	Stomach ache, diarrhoea	Antidiarrhoeal and anti-inflammatory activity [86].	Yes
*Pouzolzia bennettiana*	Stomach disorder	No relevant report found	
*Plantago major*	Wounds, inflammations	Anti-inflammatory, wound healing, anti-microbial, anti-tumor [87].	Yes
*Rhododendron arboreum*	Dysentery, diarrhoea	Quercetin, rutin and coumaric acid [53]. Protective effect against carbon tetrachloride-induced hepatotoxicity in experimental models [88].	No
*Solanum xanthocarpum*	Dental problem	No relevant report found	
*Solanum indicum*	Anthelmintic	Cytotoxic and novel compounds [89].	No
*Solanum torvum*	Anthelmintic	Anthelmintic activity of botanical extracts [90].	Yes
*Saccharum officinarum*	Jaundice	Sugar cane contains phenolic acids, flavonoids and other phenolic compounds [91].	No
*Spilanthes oleracea*	Stop bleeding, gastritis	No relevant report found	
*Zingiber officinale*	Cough, Stomachache	Antibacterial activity [92].	Partial

### Traditional knowledge secrecy and method of crude herbal medicine preparation

A total of 50 plant species belonging to 29 families and 39 genera were reportedly used by the Monpa ethnic group in their daily life. One hundred twenty-four informants (91 male and 33 female individuals) were interviewed in the study area with their age ranged between 20-60 years. Large number of informants reported that most ailments were treated at a household level. On average, significantly higher numbers of medicinal plants were claimed by illiterate village men than women (91 (73.4%) men; 33 (26.6%) women; aged between 40 and 60 years). Ethno-pharmacological survey work in India also indicated that information on the medicinal uses of plants was being confined mostly to elderly people (above 40 years of age) [46,47]. Literate people in the study area reported less number of medicinal plants as compared to illiterate ones which could probably be due to higher influence of modernization on the former. This observation holds true for related studies conducted throughout the world [48-50]. However, a study conducted by the Fassil [51] in the Northwestern Ethiopia, revealed that there was no significant difference in medicinal plant knowledge between men and women. Twenty-one male respondents (aged between 48-60 years) constitute knowledgeable, whose tradition of healing practices are revered and trusted in the local community and play multiple roles as spiritual guides and healers. Many ailments have been diagnosed and treated at household or family level and the fact that most treatments are given at household level was also reflected in the findings of other works [52-56]. There was high agreement among informants that transfer of knowledge to people outside the family circle took place on substantial payment. Most informants reported that knowledge was formally transferred along the family line and mainly through sons [57-62]. Remedy preparations often involved some sort of spiritual or ritual procedures. Ethno-pharmacological survey work conducted elsewhere demonstrated similar results [[13,45], and [46]]. This is also evident from Ethiopia where parents prefer to pass their traditional medical knowledge secrecy more to sons than to daughters [47]. Nearly 90% of informants reported that vertical transfer of medicinal plant knowledge was not taking place effectively due to lack of interest by the younger generation to learn and practice it mainly due to acculturation. It was also revealed that some informants ceased to practice traditional medicine due to the increasing availability of allopathic medicines. Informants in the study area confirmed that, medicinal plants are generally collected from different habitats. The method of preparation was mostly a hot water decoction in case of plants being administered orally and usually prepared from freshly collected plant material just before use. Studies conducted elsewhere [40,47] also revealed the frequent use of fresh materials. Fresh materials are also preferred to dried ones when they contain volatile oils, the concentration of which could deteriorate on drying. The majority of remedies were administered topically or external (16 species) or hot water decoction or oral administration (9 species), boiled vegetable (12 species), and eaten raw (9 species, see Table 2). Remedies were mostly processed using locally made mortar and pestle or grinders. Plant material used for preparation of herbal remedies was difficult to quantify but was indicated approximately 40-50 g fresh plant material or 20-25 g of powdered plant material in 300 ml of hot water to be taken twice daily after meal. Doses were mainly taken twice a day because most people were present at home on the morning and evening. The dosage depends on the age and physical appearance of the individual whilst children's were given less than adults which approximate to 100-150 ml twice daily depending on the type of illness and treatment. There were no reports of side effects following administration of herbal remedies as informed by the treated patients in particular and the local practitioners. Treatment was supposed to be continued until recovery. When patients did not show any sign of improvement after the completion of treatments with herbal remedies, they were taken to a nearby modern health centers for further examination by the physician. The ethno-botanical knowledge of Monpa ethnic group gathered in this study has been categorized and described briefly in the following sub-headings.

### Edible plants used as vegetables

The Monpa community derives common vegetables either alone or in combination from underexploited plant species like *Alocasia indica *(Roxb.), *Dioscorea alata *L., *Ipomoea batatas *(leaves and tuber) L., *Mannihot esculentum *Crantz, *Momordica charantia *L., *Phaseolus vulgaris *L., *Pouzolzia bennettiana *Wight, *Diplazium esculentum *(Retz.) Sw., *Centella asiatica *L., *Houttuynia cordata *Thunb. (green salad), *Gynura crepedioides *(BTH.) Moore (green salad), *Spilanthus oleraceae *Murr., *Litsea cubeba *(Lour) Pers. (spice), *Clerodendron viscosum *Vent., *Solanum indicum *L. (green salad), *Solanum torvum *Sw. (Green salad), *Solanum etiopicum*, *Allium sativum *Linn., and *Allium hooleri *L. (green salad). These plant species are generally sold in the local market at reasonable price. The tender shoots of selected bamboo species like *Dendrocalamus hamiltonii *Hook. f. collected in bulk was prepared by cutting it into strips or pieces and boiled. The boiled shoots are chopped finely and packed in jars, bamboo tubes (*Chunga*) or even in plastic buckets and was kept for 5-10 days for fermentation. After fermentation, the taste of chopped shoots becomes sour. Fresh bamboo shoots and its fermented products were sold in the local market as edible foodstuffs. Important domestic uses concerned presently cultivated species of *Livistona jenkensiana *Griff. used for thatching and *Bambusa tulda *Roxb. and *B. pallida *Munro., for house building work. Traditional dyeing of clothes and food items were derived from plant species namely *Illicium griffithii *Hook. and *Rubia cordifolia *L. These plant species are currently cultivated in the gardens to meet regular use due to their less accessibility. Some of the plant parts used as a food source was also ingested as a remedy: *Clerodendron colebrookianum *(blood pressure), *Momordica charantia *(diabetes mellitus), *Lindera neesiana*, *Solanum etiopicum*, and *Solanum indicum *(intestinal parasitic worms like round and tape worms

### Religious or ritual plants

The Monpa community in the eastern Himalayan province of Arunachal Pradesh follow Mahayana sect of Buddhism and are famous for hand-made paper for writing religious scripts in Monasteries (locally called "Gonpa") from stem bark of *Daphne papyracea *(Thymelaeaceae) Wall. Flowering twigs of *Thysanolaena maxima *has traditionally been used for broom making and to support the cotton wick to offer daily butter lighting in monastery. However, under the influence of modern society, today *D. papyracea *was not being used for making paper for writing Buddhist scripts as revealed during field survey work. *Gymnocladus assamicus *ripe pods are soaked in water and used to rubbed palms when preparing *torma*, a kind of sweet made of rice flour offered to the Lord Buddha during various festival ceremonies at Monasteries. The Monpa houses are built of stones and timber decorated with a small altars and chapels with statues of Lord Buddha. Offering water in little seven cups and burning butter lamps and herbal incense sticks with few herbal leaves (*Pinus wallichiana *A.B. Jackson, *Pinus longifolia *Roxb. and *Thuja occidentalis *L.) (Cupressaceae) are daily rituals. These plant species are cultivated within the premises of monasteries for their use during religious festival called Choskar. It was of common belief that burning of such herbal leaf create clean and refreshing atmosphere inside the Gonpa. The heart wood of pine tree was used for lighting of the street at night and ignition of firewood at home in olden days where there was no supply of electricity and kerosene. The resins extracted from pine wood are used as adhesives. Bulbs of *Allium sativum *and *Allium hookeri *are used in rituals and to protect against the evil spirit. A leaf of banana has long been used during festival ceremony in monastery to offer foodstuffs to local participants. The rhizomes of *Zingiber officinale *and *Mannihot esculentum *remains an integral component of daily rituals among the Monpas religious life.

### Local beverage (local beer) plants

The traditional consumption of a variety of alcoholic beverages since time immemorial is still an integral part of different ethnic communities in the north-eastern region of India. Popular traditional beer, locally known as "bhangchang", was prepared from rice (*Oryza sativa *L.), finger millet (*Eleusine coracana *Geartn.), maize (*Zea mays *L.) and buckwheat (*Fagopyrum esculentum *Moench). "Bhangchang" has traditionally been used and served in all festive occasions, birth and marriage ceremonies. A diverse knowledge system exists among the Monpa women to prepare the nutritionally rich foods and fermented beverages, which play an important role in their day to day socio-cultural and spiritual occasions. A Monpa woman uses some of the wild plants as anti-microbial and they believed that these plants are responsible for the healthy growth of yeast during the process of fermentations. During the field study, we have documented the use of young leaves and twigs of certain species like *Piper betle*, *Solanum indicum*, *Buddleja asiatica *and *Hedyotis scandens *as common growth supplements during the preparation of fermentation starter cultures containing brewer's yeast (locally called *phamzas*). The most frequently cited species were *Buddleja asiatica *and *Hedyotis scandens*. They believed that consumption of rice beer is good for health and act as a remedy for various ailments may be attributed to medicinal properties of the herbs used in the preparation of starter cultures.

### Ethno-veterinary plants

A few plants were used to improve the health state and growth of livestocks. The leaves of *Cannabis sativa *was given to the cattle and goat to cure dysentery and diarrhoea (These diseases were identified by the presence of watery stool and blood). The stem of wild *Musa paradisica *L., was regularly given to cattle particularly during pregnancy to enhance the yield of milk. A paste powder obtained from the whole plant of *Plantago major *and *Ageratum conyzoides *are commonly tied to the affected portions of cattle and goat to relieve from severe pain and inflammations. *Gymnocladus assamicus *ripe pods are soaked in water and used as disinfectant for cleaning wounds and parasites like leeches and lice on the skin of livestocks. The fully ripe pods soaked in water are used as soap for bathing because it does not cause harm to soft skin and burning sensation to eyes. Leaves are used as green manure in agricultural field crops. *Gymnocladus assamicus *is a critically rare and endangered plant species and also endemic to the north-eastern region of India [29].

### Ichthyotoxic and fish feed plants

Community seasonal fishing and hunting are of great economic activities of many tribal people including Monpa ethnic group in addition to agriculture. The study revealed a wealth of indigenous knowledge and procedures related to poison fishing with the aid of poisonous plants. This easy and simple method of fishing are forbidden in urban areas but still practiced in remote tribal areas. The active ingredients were released by macerating the appropriated plant parts with the help of wooden stick or hammer, which were then introduced into the water environment. Depending upon time and conditions, the fish begin to float to the surface where they can easily be collected with bare hand. A total of seven plant species, namely *Castanopsis indica*, *Derris scandens*, *Aesculus assamica*, *Polygonum hydropiper*, *Spilanthes acmella*, *Ageratum conyzoides*, and *Cyclosorus extensus *were used to poison fish during the month of June-July every year and leaves of three species like *Ipomoea batatas*, *Mannihot esculenta*, and *Zea mays *were used as common fish foodstuffs. The two main molecular groups of fish poisons in plants (the rotenones and the saponins) as well as a third group of plants which liberate cyanide in the water account for nearly all varieties of fish poisons [63]. The underground tuber of *Aconitum ferrox *was widely used in arrow poisoning to kill ferocious animals like bear, wild pigs, gaur and deer. The killing of Himalayan bear was very common practice among the tribal people and the gall bladders are highly priced in the local market. The dried gall bladders of bear are given orally in low doses to cure malaria since ancestral times.

## Conclusions

This ethno-botanical survey results probably revealed the rich wealth of indigenous knowledge and usage custom of traditional plants associated with rural people of Arunachal Pradesh. Despite their use in traditional medicines, plant species documented in the present field work have been extensively used for improving the health of livestock, fish foodstuffs, ethno-fishing technology, local fermentation technologies, religious and food plants as well. There was no written document of traditional healing knowledge and transmission to the future generation take place only through oral communication. The immediate and serious threat to the local medical practice in the study area seems to have come from the increasing influence of modernization, deforestation due to anthropogenic activities and migration of the younger generations to urban areas leaving a gap in the cultural beliefs and practices of indigenous society. However, there was a potential threat to the medicinal flora of the area as a result of the increasing trend of shifting cultivation (annual clearing of forest) and cultural changes signaling the need for serious efforts to create public awareness so that the appropriate measures are taken to conserve the suitable environments required to protect the medicinal plants in the natural ecosystems. More detailed ethnopharmacological investigations need to be conducted in this area particularly in regard to conservation strategies and sustainable use of medicinal plants.

## Competing interests

The authors declare that they have no competing interests.

## Authors' contributions

NDN developed and designed the research study and conducted field survey work data and wrote the manuscript. ST helped in preliminary identification of plant species and corrected the manuscript. MM and SCM read, supervised and approved the final manuscript. All authors have read and approved the final manuscript.
